# NKG2A/CD94 Is a New Immune Receptor for HLA-G and Distinguishes Amino Acid Differences in the HLA-G Heavy Chain

**DOI:** 10.3390/ijms21124362

**Published:** 2020-06-19

**Authors:** Gia-Gia T. Hò, Alexander A. Celik, Trevor Huyton, Wiebke Hiemisch, Rainer Blasczyk, Gwendolin S. Simper, Christina Bade-Doeding

**Affiliations:** 1 Institute of Transfusion Medicine and Transplant Engineering, Hannover Medical School, 30625 Hannover, Germany; Ho.Gia-Gia@mh-hannover.de (G.-G.T.H.); alexander.celik@outlook.com (A.A.C.); Hiemisch.Wiebke@mh-hannover.de (W.H.); Blasczyk.Rainer@mh-hannover.de (R.B.); Simper.Gwendolin@mh-hannover.de (G.S.S.); 2 Max Planck Institute for Biophysical Chemistry, 37077 Göttingen, Germany; trevor.huyton@mpibpc.mpg.de

**Keywords:** HLA-G, NKG2A, NK cells

## Abstract

Natural killer (NK) cell therapies are a tool to antagonize a dysfunctional immune system. NK cells recognize malignant cells, traffic to a tumor location, and infiltrate the solid tumor. The immune checkpoint molecule human leukocyte antigen (HLA)-G is upregulated on malignant cells but not on healthy surrounding cells, the requirement of understanding the basis of receptor mediated events at the HLA-G/NK cell interface becomes obvious. The NK cell receptors ILT2 and KIR2DL4 have been described to bind to HLA-G; however, their differential function and expression levels on NK cell subsets suggest the existence of an unreported receptor. Here, we performed a ligand-based receptor capture on living cells utilizing sHLA-G*01:01 molecules coupled to *TriCEPS* and bound to NK cells followed by mass spectrometric analyses. We could define NKG2A/CD94 as a cognate receptor of HLA-G. To verify the results, we used the reciprocal method by expressing recombinant soluble heterodimeric NKG2A/CD94 molecules and used them to target HLA-G*01:01 expressing cells. NKG2A/CD94 could be confirmed as an immune receptor of HLA-G*01:01. Despite HLA-G is marginal polymorphic, we could previously demonstrate that the most common allelic subtypes HLA-G*01:01/01:03 and 01:04 differ in peptide repertoire, their engagement to NK cells, their catalyzation of dNK cell proliferation and their impact on NK cell development. Continuing these studies with regard to NKG2A/CD94 engagement we engineered recombinant single antigen presenting *K562* cells and targeted the surface expressed HLA-G*01:01, 01:03 or 01:04 molecules with NKG2A/CD94. Specificity and sensitivity of HLA-G*01:04/NKG2A/CD94 engagement could be significantly verified. The binding affinity decreases when using K562-G*01:03 or K562-G*01:01 cells as targets. These results demonstrate that the ligand-receptor assignment between HLA-G and NKG2A/CD94 is dependent of the amino acid composition in the HLA-G heavy chain. Understanding the biophysical basis of receptor-mediated events that lead to NK cell inhibition would help to remove non-tumor reactive cells and support personalized mild autologous NK cell therapies.

## 1. Introduction

The non-classical human leukocyte antigen (HLA) molecule HLA-G is a mediator of immune tolerance, its expression is restricted to certain tissues and distinct health conditions. HLA-G is expressed in pregnancies by cells of the trophoblast at the maternal–fetal interface [1,2]; in preeclampsia and recurrent spontaneous abortion the expression levels of HLA-G are decreased [3,4,5]. HLA-G expression could be determined in immune privileged organs as for example the cornea [6], the thymus [7,8], in erythroid and endothelial precursors [9], and in serum/plasma samples of healthy subjects produced by activated CD14^+^ monocytes [10,11]. Upregulation of HLA-G is seen in viral infections [12], cancer (glioblastoma [13,14], melanoma [14,15], breast and ovarian cancer [14], acute leukemia [16]), and other immune-mediated diseases [17,18]. Expression of HLA-G following transplantation is associated with a reduced rate of rejection [19], indicating the importance of HLA-G for the reliability of the human body in exceptional circumstances such as pregnancy, cancer, or transplantation.

The HLA-G locus encodes for 19 distinct proteins [20]. Despite being rather invariable in comparison to class Ia molecules, HLA-G molecules are capable of presenting diverse set of peptides [21,22,23,24]. The most prevalent allele in Europe by far is HLA-G*01:01, followed by HLA-G*01:04, HLA-G*01:03, and the null allele HLA-G*01:05N [25,26,27].

In its function of mediating immune tolerance, HLA-G interacts with different immune effector cells such as natural killer (NK) cells, T and B cells, monocytes, and dendritic cells (DCs) [28]. By binding their inhibitory receptors, the immune effector function is disabled and immune inhibition is caused [28]. At least five receptors are described to bind HLA-G: ILT2, ILT4, KIR2DL4, CD8, and CD160 [29] (see Table 1). Interacting with these receptors, HLA-G can be helpful as in prevention of rejection of the fetus or transplants or it can be fatal when masking malignancies [30,31].

ILT2 (LILRB1) is expressed by NK cells, T cells, B cells, DCs, and decidual macrophages [29,32,33] while ILT4 (LILRB2) expression is restricted to monocytes, macrophages and DCs [29]. Both ILT2 and ILT4 have inhibitory effects on the immune response [34]; interacting with HLA class I molecules in general, the affinity for HLA-G is much higher as shown by surface plasmon resonance [35].

The NK cell receptor KIR2DL4 recognizes HLA-G [36], however due to internalization KIR2DL4 is only temporarily localized on the surface of cells, implying that mainly soluble HLA-G interacts with this receptor [29].

While ligation between HLA-G and a TCR has never been detected [34], HLA-G engagement with CD8 receptors on NK and T cells could be demonstrated; the HLA-G/CD8 ligation leads to the induction of apoptosis via FasL upregulation [37]. CD8 competes with ILT2 and ILT4 for binding to HLA-G [35]. Expression of HLA-G leads to the upregulation of ILT2, ILT3, ILT4, and KIR2DL4 [38], possibly leading to an increased threshold for the activation of the immune system. Additionally, CD160 on endothelial cells was found bind to soluble HLA-G1 (sHLA-G1) resulting in the inhibition of angiogenesis [39].

It still remains to be ascertained how HLA-G has the ability to modulate the immune system by a subtle interplay between HLA expression, peptide presentation and immune receptor recognition. To date it is known for example that activated CD8^+^ T cells are killed by apoptosis following interaction with HLA-G [40]. Specific lysis through KIR2DL4-expressing NK cells can be blocked by expression of HLA-G [36] and maturation of ILT4-positive DCs can be interrupted [41]. In vitro HLA-G expression by tolerogenic DC-10 is required for differentiation of type 1 T regulatory cells [42].

Another non-classical HLA-Ib molecule, HLA-E, that is upregulated during pathogenic episodes and stabilized through binding to the HLA-G leader peptide VMAPRTLFL constitutes a ligand for the inhibitory NKG2A/CD94 receptor [43]. Biophysical and structural studies determined the binding of HLA-E bound to the leader peptide of HLA-Cw*07 (VMAPRALLL) and the HLA-E^VMAPRTLFL^ complex [44] engaged with NKG2/CD94 receptors [45,46]. In 2008, Petrie et al. could impressively comprehend the structural basis of the HLA-E^VMAPRTLFL^/NKG2A/CD94 interface and explain the inhibitory immune response as a result of pathogenic immune escape mechanism. Earlier studies on NKG2A/CD94 as a potential receptor for HLA-G molecules [47,48] could not exclude the presence of HLA-E^VMAPRTLFL^ molecules on the surface of HLA-G expressing cells. NKG2A/CD94 could therefore not be implemented in the list of certain receptors for HLA-G molecules [49].

NK cell therapies are a tool to antagonize downregulation of HLA-Ia molecules during pathogenic episodes [50]. NK cell receptor recognition of HLA-Ia absence on leukemic cells and HLA-G upregulation on those HLA-Ia empty cells is a walk on a tightrope for a diseased immune system [28]. The insusceptibility of some patients to those therapies suggests an unknown inhibitory NK cell receptor. For personalized NK cell-based immunotherapies it is imperative to comprehensively assess the role of HLA-G. The immune checkpoint molecule HLA-G is upregulated on malignant cells, where it presents an altered peptide repertoire [51], but not on healthy surrounding cells, the requirement of understanding the basis of receptor mediated events at the HLA-G/NK cell interface becomes obvious.

We used the *TriCEPS* ligand-based receptor capture technology to evaluate receptors for sHLA-G*01:01 on NK cells. The experiments are performed under almost physiological conditions on living cells allowing for the detection of receptors for a certain ligand. The *TriCEPS* molecule is comprised of three domains enabling (a) ligand binding, (b) receptor binding, and (c) detection and purification. The aim is to detect an unreported NK cell receptor for HLA-G and to analyze the magnitude of HLA-G allelic variants on receptor ligation.

## 2. Results

### 2.1. The TriCEPS Method Can Be Performed Using NKL Cells as Target Cells and sHLA-G*01:01-TriCEPS as Ligand

**Figure A1:**
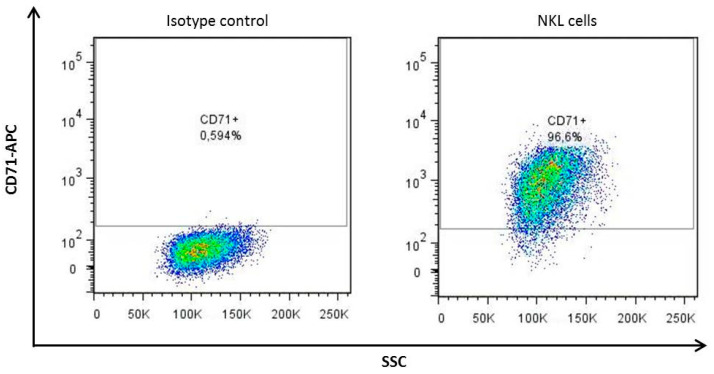
The transferrin receptor TRF1 (CD71) is expressed on the cell surface of *NKL* cells. Presence of the transferrin receptor was checked in order to use this receptor as positive control. Measurement was performed in duplicates; one representative is shown.

In order to perform a ligand-based receptor capture several pretests had to be applied. Since transferrin was planned to be used as a positive control, the suitability of the cell line was verified by measuring the expression of the transferrin receptor TFR1 (CD71) on the cell surface of NKL cells. It could be shown that the transferrin receptor TFR1 (CD71) is highly expressed on NKL cells (Figure A1). The experiment was performed in duplicates exhibiting levels of at least 96% for CD71^+^ cells.

**Figure A2:**
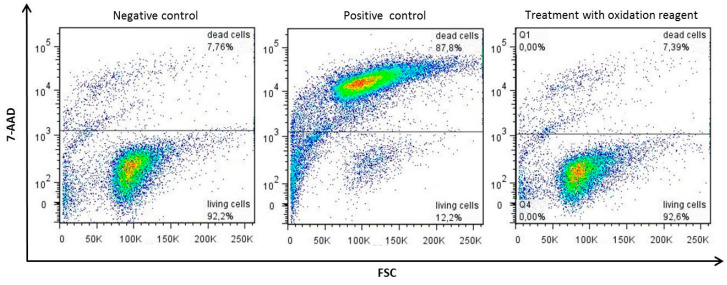
Oxidation does not impair the viability of NKL cells. The impact of the mild oxidation of on the viability of the target cells was validated. NKL cells were treated with 1.5 mM NaIO4 oxidation reagent for mild oxidation. Dead cells are stained with 7-AAD.

Furthermore, the effect of oxidation on the viability of the *NKL* cells was ascertained. Following treatment of the target cells with 1.5 mM NaIO4 oxidation reagent no impact on the survival of *NKL* cells was observed (Figure A2).

**Figure A3:**
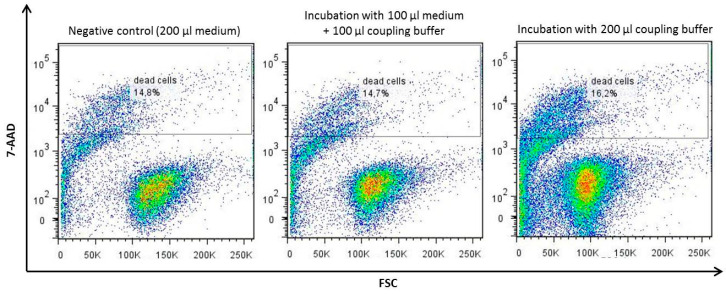
The viability of *NKL* cells is not impaired by the coupling buffer. The effect of incubation with the coupling buffer on the viability of the target cells was examined. Dead cells are stained with 7-AAD.

Additionally, the viability of *NKL* cells post treatment with coupling buffer was determined. The staining with 7-AAD showed that compared to the negative control, the number of dead cells in the samples did not increase after incubation with the coupling buffer (Figure A3). 

**Figure A4:**
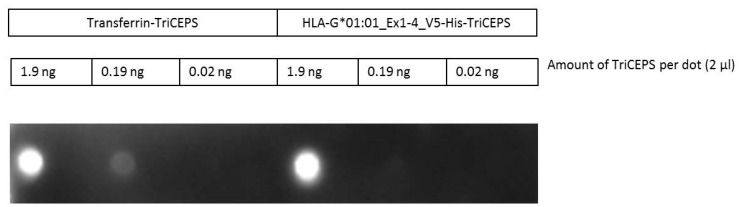
Coupling with sHLA-G and TRF1 was successful. The positive control transferrin and the ligand of interest sHLA-G*01:04 were coupled to the *TriCEPS* molecule. The success of the coupling reaction was observed by performing of a dot blot. Only coupled *TriCEPS* is able to diffuse into the membrane. The dot blot was performed in 3 different dilutions: 1.9 ng *TriCEPS*/2 µL, 0.19 ng *TriCEPS*/2 µL, and 0.02 ng *TriCEPS*/2 µL.

A dot blot was used to validate coupling of HLA-G and transferrin as positive control to *TriCEPS*. Unlike the proteins that serve as ligands, uncoupled *TriCEPS* is not able to diffuse into the membrane. Thus, the biotin domain of *TriCEPS* can only be detected on the blot if it has bound the ligand, thereby confirming the success of the coupling reaction. Successful ligand-*TriCEPS* coupling was achieved (Figure A4).

**Figure A5:**
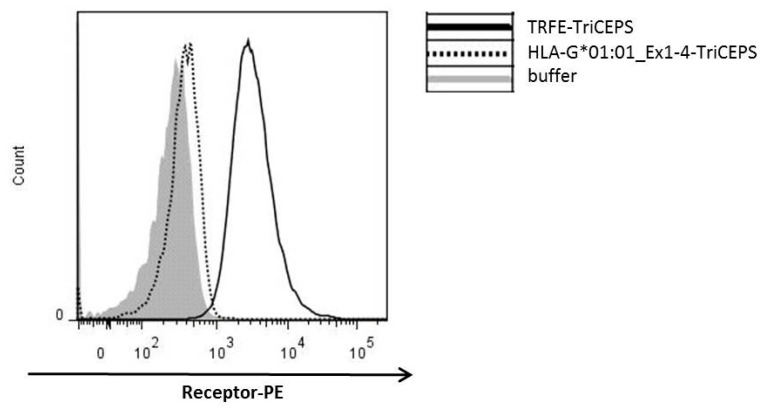
Binding of ligand-*TriCEPS* to NKL cells was successful. The positive control transferrin and the ligand of interest sHLA-G*01:04 were coupled to the *TriCEPS* molecule and incubated with the target cells. Via detection of the third domain of the *TriCEPS* molecule, a biotin domain, successful binding of the coupled ligands was observed using flow cytometry.

To verify whether binding of the ligands was possible, *NKL* target cells were incubated with undiluted ligand-*TriCEPS* molecules. When incubation was performed at 4 °C for 2 h, as indicated in the manual, the cells treated with sHLA-G*01:01-*TriCEPS* only coupling of the positive control was successful. The experiment was repeated with increased an incubation temperature of 37 °C and a prolonged incubation time of 4 h. Under these conditions binding of sHLA-G*01:01-*TriCEPS*, as well as binding of transferrin-*TriCEPS* was observed (Figure A5). Nearly all cells were positive for transferrin-*TriCEPS*, whereby the fraction of sHLA-G*01:01-*TriCEPS*-positive cells was much smaller.

*NKL* cells can be used as target cells and sHLA-G*01:01-*TriCEPS* as ligands for LCR-*TriCEPS* receptor capture.

### 2.2. NKG2A as a Potential Receptor for HLA-G 

**Figure 1:**
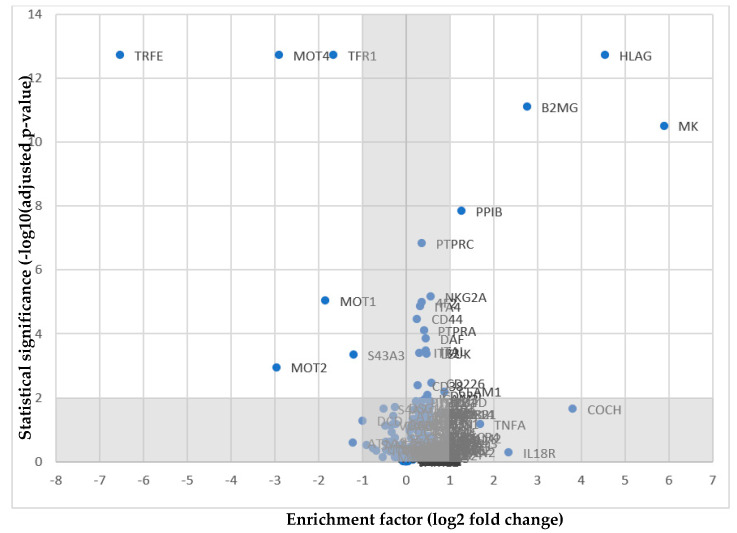
Ligand-based receptor capture was performed using *NKL* cells as target cells in order to identify receptors for sHLA-G. The *TriCEPS*-construct was bound to the ligand of interest sHLA-G or to the positive control transferrin before binding of the coupled molecules to periodate-oxidized NKL cells. The cells were lyzed, proteins digested and the *TriCEPS*-construct was purified. After the enzymatic peptide release the peptides were analyzed via mass spectrometry. The results of the final *TriCEPS* experiment are depicted in a volcano plot showings the enrichment of possible receptors for the positive control transferrin (left) and the ligand of interest HLA-G (right). On the y-axis the statistical significance is indicated (−log10 (adjusted *p*-value)). On the x-axis the enrichment factor (Log2 fold change) between the two conditions is shown. A statistical significance of at least 2 and an enrichment factor of 2 fold and greater is required.

The ligand of interest sHLA-G or the positive control transferrin was coupled to the *TriCEPS*-construct. Using periodate *NKL* target cells were mildly oxidized. The receptor capturing in the main experiment was performed for 30 min at 37 °C in the presence of a catalyzer provided by Dualsystems Biotech. Cell lysis was performed and proteins were digested, the *TriCEPS*-construct was purified using the third domain. Peptides were released enzymatically and were analyzed via mass spectrometry. The volcano plot shows the results of the analysis with the significance plotted against the fold-change (Figure 1). Four proteins were found with a significance of 2 or greater and a *p*-value less or equal to 0.01 (Table 2). HLA-G and β2m were identified as components of the ligand itself. Receptor candidates midkine and peptidyl-prolyl cis-trans-isomerase both have an enrichment factor of 2 or greater and a *p*-value less or equal to 0.01, but are soluble proteins without transmembrane domain. But with an enrichment factor of 0.5643 and a significance of 51,590 NKG2A was identified as alternate candidate, although the fold-change is below the threshold. Different new receptor candidates for HLA-G could be identified utilizing LCR-*TriCEPS* receptor capture. One potential new receptor candidate for HLA-G is the inhibitory NKG2A/CD94 receptor.

### 2.3. NKG2A/CD94 Distinguishes AA Differences in the HLA-G Heavy Chain

**Figure 2:**
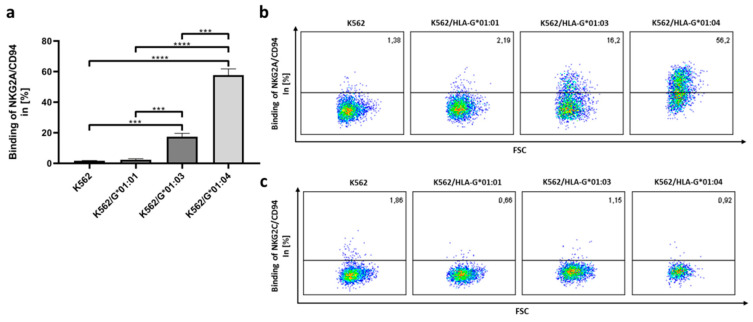
NKG2A/CD94 distinguishes amino acid (AA) differences in the HLA-G heavy chain. (**a**) K562/HLA-G*01:01, K562/HLA-G*01:03, or K562/HLA-G*01:04 and *K562* cells were analyzed for NKG2A/CD94 binding. Cells were incubated with sNKG2A/CD94 coupled to *V5-His-tag*. Binding was detected by an anti-V5 antibody and a PE labelled secondary antibody. Experiment was performed in triplicates (*n* = 3). Significance of (***) describes a *p*-value of *p* < 0.001 and significance of (****) describes a *p*-value of *p* < 0.0001; (**b**) Representative FACS plots for the binding of sNKG2A/CD94 and (**c**) sNKG2C/CD94. Depicted numbers in each plot indicate the binding of NKG2/CD94 in percentage.

Utilizing the *TriCEPS* technology, we could define NKG2A/CD94 as a cognate receptor of HLA-G. To verify the results, we performed the reciprocal experiment by expressing recombinant soluble heterodimeric NKG2A/CD94 (sNKG2A/CD94) molecules and used them to target HLA-G*01:01, HLA-G*01:03 or HLA-G*01:04 expressing cells; recombinant soluble NKG2C/CD94 (sNKG2C/CD94) and non-transfected *K562* cells served as negative control (Figure 2).

By comparing the inhibitory NKG2A/CD94 and the activating NKG2C/CD94 receptors, exclusively the inhibitory receptor NKG2A/CD94 could be confirmed to bind to HLA-G molecules. No binding of sNKG2/CD94 could be detected in the non-transfected control. However, the binding of NKG2A/CD94 illustrated variability depending on the HLA-G allelic variant. Among HLA-G allelic variants, the NKG2A/CD94/HLA-G*01:04 engagement showed the strongest binding affinity with 57.7%. The binding affinity decreases when using K562/HLA-G*01:03 cells to 17.4%.

NKG2A/CD94 and HLA-G engagement could be verified in the reciprocal experiment. NKG2A/CD94 distinguished AA differences in the HLA-G heavy chain and preferentially binds HLA-G*01:04.

## 3. Discussion

The role of HLA-G is not fully understood, yet. HLA-G plays a crucial role in protecting the fetus as semi-allogenic transplant from rejection [3,4,5]; in cancer as well as in transplant acceptance HLA-G seems to be a key player. The distinct interaction of HLA-G with immune receptors remains unclear. Until now ILT2, ILT4, KIR2DL4, CD8, and CD160 have been identified as receptors for HLA-G. None of them are known to distinguish between HLA-G alleles. With regard to personalized NK cell therapies to overcome tumor evasion strategies, the knowledge of individual ligand-receptor interfaces is fundamental.

Earlier, the inhibitory receptor NKG2A/CD94 was discussed as receptor for HLA-G [47,48], but was discarded later on due to co-expression of HLA-E on the cell lines used [49]. In this study we performed a method allowing receptor capture on living cells under almost physiological conditions as shown by Frei et al. [62,63]. This methodology enables the identification of receptors for known ligands without the need for genetic manipulation. Additionally, it is possible to not only detect stable, but also transient interactions. Using the LCR-*TriCEPS* technology we could confirm NKG2A/CD94 as new receptor for HLA-G. The NKG2A/CD94 heterodimer is a member of the family of C-type lectin-like receptors and suppresses NK cell activation. In previous studies we could define HLA-G allelic variants to differentially modulate tolerance of immune cells. Other hits in the experiment were soluble proteins as midkine, a heparin-binding growth factor that is overexpressed in many tumors and leukemia [64]. NKL cells are a leukemia cell line [65], thereby proteins as midkine might be strongly expressed and potentially bound as peptides to HLA-G. For comprehension, soluble proteins were excluded from the results of potential receptors; since the aim was to find an NK cell bound receptor that could be targeted by HLA-G.

The reciprocal experiment using sNKG2A/CD94 heterodimers bound to cells expressing HLA-G variants was therefore utilized to analyze the potential of NKG2A/CD94 to differentially engage with HLA-G*01:01/01:03/01:04. The innate immune receptor NKG2A/CD94 showed unambiguously the ability to distinguish AA differences in the HLA-G heavy chain. HLA-G*01:01 and G*01:04 differ in a single AA at outer loop position 110 resulting in the selection and presentation of a different peptidome, whereas G*01:03 differs in a single AA at position 31 from G*01:01 and shares the peptide binding motif with G*01:01 [66]. The affinity of NKG2A/CD94 for G*01:04 is highest, followed by G*01:03, while the NKG2A/CD94-G*01:01 binding is marginal. The distinct variants do not only differ in the features of the presented peptides, but have been shown to activate decidual NK cells in an allele-specific way with G*01:04 being the strongest catalyst [67]. Furthermore, we could previously show that G*01:04 is more protective against NK cell-mediated lysis than the other 2 allelic variants analyzed [66]. This underlies the extraordinary role of G*01:04 as mediator of immune tolerance.

Non-classical HLA molecules act as ligands for the innate immune system and are known to be oligomorphic. However, the invariable non-classical HLA molecule HLA-E has been shown to interact with receptors of the innate immune system in a competing manner depending on the sequence of the presented peptide [23,68]. Whether the engagement of NKG2A/CD94 and HLA-G is dictated by the bound peptide remains to be unraveled since this receptor engages peptide-specific with HLA-E [46,69]. HLA-G*01:01 and HLA-G*01:03 possess Proline as auxiliary anchor at peptide position p3, this could not be defined for HLA-G*01:04 derived peptides; the differential peptide features seem to have no structural impact on the allelic variants [66]. The necessity for a reliable assay to define a certain NK cell receptor that binds to peptide/HLA-G molecules becomes obvious.

This study is based on the capture of effector cell receptors on living cells. The recombinant sHLA-G molecules used as capture proteins in this study are bound to a diversity of peptides [66]. Since we could previously show that the interaction of pHLA-E:NKG2A/CD94 and pHLA-E:NKG2C/CD94 is highly dependent on the peptide that is presented by HLA-E [68]; it is unclear if the binding of HLA-G to NKG2A/CD94 might be peptide-dictated as well. However, HLA-G:NKG2A/CD94 engagement could be clearly ascertained, while binding of HLA-G to NKG2C/CD94 could not be detected using LCR-*TriCEPS* technology or binding experiments using sNKG2C/CD94. It should be kept in mind that the used sHLA-G molecules are derived from cells maintained under optimal conditions; thus, peptide presentation and selection is not influenced by stress [70]. Therefore, it remains ambiguously if NKG2A/CD94 would engage with the same affinity to HLA-G bound to peptides selected under stress or pathogenic immune settings.

NK cell-based immunotherapy against tumors has become an important field of research. NK cell function is regulated by an array of inhibitory and activating receptors of which NK cell-inhibitory receptors are specific for HLA class I molecules; alloreactive NK cells are reactive due to *missing self* [71,72]. Based on these principles NK cell therapies were developed using T- and B-cell depleted hematopoietic stem cell transplantation (HSCT). Alternatively, NK cells are activated and used for adoptive NK cell therapies using the *missing self*-principle [73], and monoclonal antibodies are used to block inhibitory checkpoints in NK cells. The immortalized NK cell line *NK-92* is in use as an intravenous infusion for tumor treatment [74,75]. The immunotherapy with *NK-92* cells is in phase 1 of clinical studies (Clinical Trial ID NCT0090809 and NCT00990717). In contrast to autologous NK cells, allogenic NK cell infusion bears the risk to develop unpredictable immune reactions. However, it remains questionable why not all tumor localizations are affected by these therapies. Due to the tumor microenvironment inhibiting immune effector functions, tumors become invisible for the immune system [76,77]. For instance, intratumoral NK cells display higher expression levels of certain receptors, including NKG2A/CD94 [78,79]. In order to eliminate non-tumor reactive cells in NK cell therapies, screening for NKG2A/CD94 is a possibility to prohibit suppression of NK cells. As already described for NKG2A/CD94 expression and HLA-E-positive tumors, downregulation or blocking of NKG2A/CD94 enhances the antitumor function of NK cell infusions [80]. This might be even more important in the light of HLA-G interacting with NKG2A/CD94 since HLA-G-expression is independent of the expression of other HLA molecules. HLA-E was found to bind NKG2A/CD94 and NKG2C/CD94 via tetramer binding to cells transfected for expression of certain NK cell receptors after having observed binding to NK cells and a subset of T cells [81]. The ratio of NKG2A/NKG2C was proposed as biomarker for disease progression in HIV infection [82]. Peripheral NK cells of healthy donors express mostly either inhibitory NKG2A or activating NKG2C [83,84,85]. It would therefore be tremendously helpful for personalized therapies to target non-tumor-reactive NKG2A/CD94 positive NK cells.

In summary, these experiments confirm and substantiate the assumption of NKG2A/CD94 to be a new receptor for HLA-G. Understanding the biophysical basis of receptor-mediated events that lead to NK cell inhibition would help to remove non-tumor reactive cells and support personalized mild autologous NK cell therapies.

## 4. Materials and Methods

### 4.1. Maintenance of the Cell Lines

HLA class I negative cell line *K562* was maintained in *RPMI 1640* (Lonza, Basel, Switzerland) supplemented with 10% heat inactivated fetal calf serum (FCS, Lonza, Basel, Switzerland), 2 mM L-glutamine (c. c. pro, Oberdorla, Germany), 100 U/mL penicillin and 100 µg/mL streptomycin (c. c. pro, Oberdorla, Germany).

*HEK293T* cells, used for production of lentiviral particles, were cultured in DMEM (Lonza, Basel, Switzerland) supplemented with 10% heat inactivated FCS, 2 mM L-glutamine, 100 U/mL penicillin, 100 µg/mL streptomycin and 1 mg/mL geneticin (Life Technologies, Carlsbad, CA, USA).

*NKL* cells were maintained in *RPMI 1640* supplemented with 15% heat inactivated fetal calf serum, 1% Natriumpyruvate (c. c. pro, Oberdorla, Germany), 200 U/mL IL-2 (Rocky Hill, NJ, USA), 100 U/mL penicillin and 100 µg/mL streptomycin.

All cell lines were maintained at 37 °C and 5% CO_2_.

### 4.2. Cloning of HLA-G Constructs

The construct encoding for HLA-G*01:01 (exon 1–6) was generated from *JEG-3* cDNA, subcloned into the lentiviral vector *pRRL.PPT.SFFV.mcs.pre* as previously described [66]. Constructs for HLA-G*01:03 and HLA-G*01:04 were generated utilizing *site-directed* mutagenesis by introducing single point mutation at position c.162A > T for HLA-G*01:03 or c.400C > A for HLA-G*01:04.

Constructs encoding for sHLA-G*01:0x were cloned into the lentiviral vector *pRRL.PPT.SFFV.mcs.pre*, as previously described [66]. The respective inserts were verified through sequencing.

### 4.3. Cloning of Plasmid Encoding for Soluble NKG2/CD94 Heterodimers

The method used for cloning of a vector encoding for sNKG2A/CD94 and sNKG2C/CD94 heterodimers is described by Pump et al. [68]. Constructs encoding for sNKG2A/CD94 or sNKG2C/CD94 with *V5-His-tag* were cloned into the lentiviral vector *pRRL.PPT.SFFV.mcs.pre*. The respective inserts were verified through sequencing.

### 4.4. Stable Lentiviral Transduction of K562 Cells with HLA-G and NKG2/CD94 Constructs

As described by Bade-Doeding et al. [86]. *HEK293T* cells were transfected with the target plasmids (10 µg/5 × 10^6^ cells) and the packaging and envelope vectors *psPAX2* and *pmD2.G* (both 5 µg/5 × 10^6^ cells) using *Lipofectamine^®^ 2000* (Life Technologies, Carlsbad, CA, USA). The lentiviral particles were utilized to transduce *K562* cells.

The expression of sHLA-G was confirmed by ELISA (coating antibody anti-HLA class I clone W6/32 (Biorad, Hercules, CA, USA) and detection antibody anti-β2m (Agilent Technologies, Santa Clara, CA, USA)) and western blot (anti-V5 (Biorad, Hercules, CA, USA)). The presence of mHLA-G on the cell surface was verified by flow cytometry (anti-human HLA-G clone 87G (Biolegend, San Diego, CA, USA)). The presence of sNKG2/CD94 was confirmed by ELISA (coating antibody anti-hNKG2A (Clone 131411, R&D Systems, Minneapolis, MN, USA) or anti-hNKG2C (Clone 134522, R&D Systems, Minneapolis, MN, USA) and detection antibody anti-V5-tag (Clone SV5-Pk1, Biorad, Hercules, CA, USA)).

### 4.5. Large-Scale Production of sHLA-G, sNKG2A, and sNKG2C 

**Figure A6:**
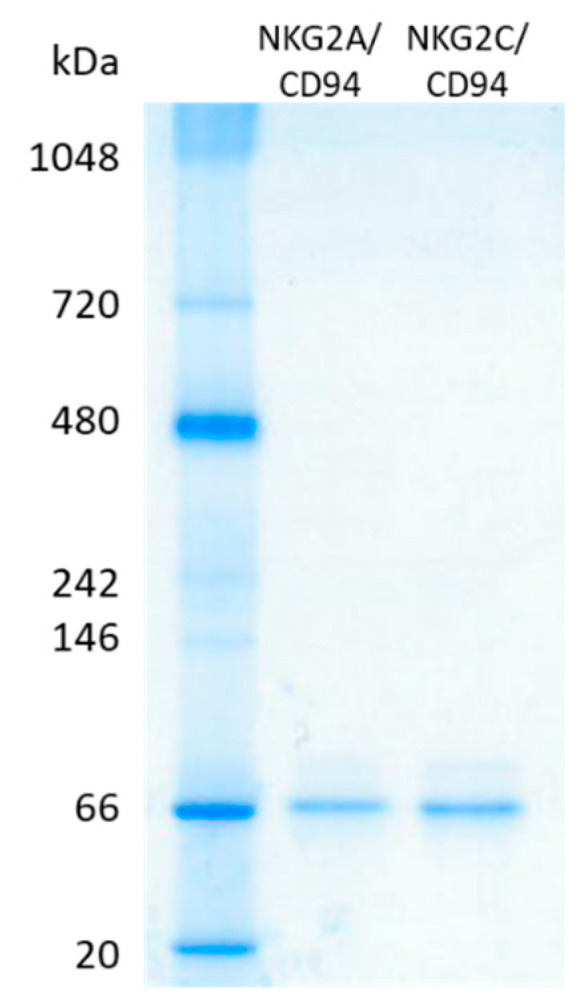
Native PAGE of purified sNKG2A/CD94 and sNKG2C/CD94. Purification of heterodimeric complex of NKG2A/CD94 and NKG2C/CD94 was examined via native PAGE. Clear bands were detected at the expected protein size.

sHLA-G*01:01, sHLA-G*01:03, sHLA-G*01:04, sNKG2A, and sNKG2C were produced in large scale according to the soluble HLA technology [87]. The cells were cultured in bioreactors (Integra Biosciences, Biebertal, Germany) at 37 °C and 5% CO_2_ for 10 days, then the cells were pelleted (300× *g*, 10 min) and the supernatant was filtered (0.45 µm (Merck, Darmstadt, Germany) prior to affinity chromatography. The sHLA-G molecules were purified at pH 8.0 using NHS-activated *HiTrap* columns coupled with w6/32. The sNKG2A and sNKG2C molecules were purified at pH 8.0 using NHS-activated *HiTrap* columns coupled with anti hNKG2A (clone 121411, R&D systems, Minneapolis, MN, USA) or anti-hNKG2C antibody (clone 134522, R&D systems). For elution 100 mM glycine buffer at pH 2.7 (adjusted with HCl) was used. The samples were neutralized by addition of 1 M Tris-HCl (pH 8.5 at 4 °C). The success of the purification of HLA-G was confirmed quantitatively via an HLA class I-specific ELISA and qualitatively via western blot. The successful purification of sNKG2A/CD94 and sNKG2C/CD94 was confirmed quantitatively via ELISA and qualitatively via native PAGE (Figure A6).

### 4.6. LCR-TriCEPS Method for Capturing of Receptors for HLA-G

In order to analyze HLA-G-receptor interactions, ligand-based receptor capturing was applied using purified sHLA-G and the NK-cell line *NKL*. The technology is based on a chemical compound called *TriCEPS* (Dualsystems Biotech AG, Schlieren, Switzerland) and allows under almost physiological conditions for recognition of receptors for an existing ligand on living cells [62,63]. This molecule comprises three domains. The first domain is an N-hydroxysuccinimide ester allowing the nonspecific coupling of the *TriCEPS* molecule to primary amines of the ligand. Aldehydes are introduced to carbohydrates of the receptor via mild oxidation, to facilitate binding of the *TriCEPS* molecule to the receptor. This enables the second domain of the *TriCEPS* molecule, a hydrazine group, to react with the aldehydes and thus to permanently bind to the receptor. The third domain is necessary to purify the *TriCEPS* molecule and all bound components before mass spectrometric analysis.

The experiment was performed using the *TriCEPS* kit provided by Dualsystems Biotech AG. Pretests and the final binding reaction were conducted and then sent to Dualsystems Biotech AG for cell lysis, protein digestion, purification of the *TriCEPS*-molecule, peptide release, and the final mass spectrometric analysis. The receptor capturing during the final reaction was performed in the presence of a catalyzer.

### 4.7. Detection of sNKG2A/CD94 Binding to HLA-G

**Figure A7:**
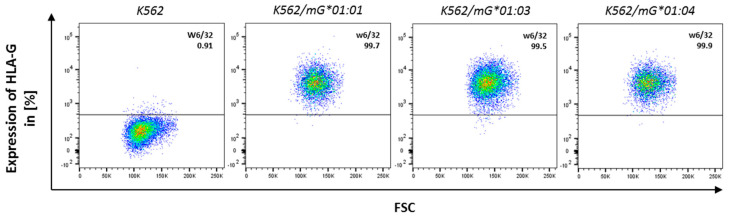
FACS analysis of HLA-G expression. Transduction efficiency of HLA-G*01:0x was verified by FACS analysis. Untransduced K562 cells were used as negative control.

**Figure A8:**
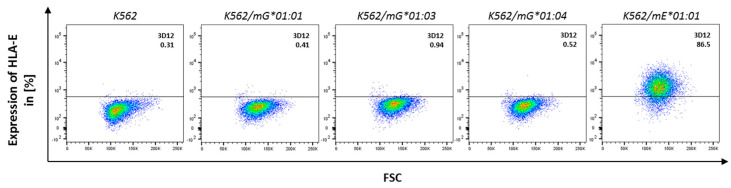
FACS analysis of HLA-E expression in HLA-G transduced *K562* cells. Transduced cells were analyzed with an anti-HLA-E antibody (clone 3D12) for the presence of HLA-E on the cell surface. *K562* cells served as negative control and transduced K562/mE*01:01 cells served as positive control.

A flow cytometry-based assay was used to test whether recombinant sNKG2A/CD94 can bind to recombinant membrane bound HLA-G on *K562* cells. Each experiment was performed using three technical replicates. *K562* cells were lentivirally transduced with vectors encoding for HLA-G*01:0x variants and subsequently sorted for equal HLA-G expression as described by Celik et al. [66] (Figure A7). To exclude cross reaction with HLA-E on the cell surface, recombinant *K562* cells were analyzed for the expression of HLA-E molecules (Figure A8). For all tests, 1 × 10^6^ HLA-G*01:0x presenting cells were incubated with 200 nM of purified sNKG2A/CD94 or 200 nM of purified sNKG2C/CD94 in case of control for 2 h at 37 °C. Non-transduced *K562* cells served as negative control. For detection of sNKG2/CD94, cells were incubated with anti-V5-tag antibody for 30 min at 4 °C. Afterwards, cells were incubated with goat-anti-mouse PE-coupled secondary antibody (BD Bioscience) for 30 min at 4 °C in the dark for detection.

## Figures and Tables

**Table 1:** Receptors for human leukocyte antigen (HLA)-G.

	Cells	HLA-G	Method
ILT2	NK cells, T cells, DCs, and decidual macrophages	sHLA-G1	Crystal structure [52]
		sHLA-G1	Crystal structure [53]
		sHLA-G1	Surface plasmon resonance [35]
		sHLA-G1	Surface plasmon resonance [54]
		HLA-G tetramers	Tetramer-binding assays [55]
		mHLA-G1	Cytotoxicity assays [33]
ILT4	monocytes, macrophages, and DCs	β_2_m-free HLA-G1 dimers	Surface plasmon resonance [52]
		sHLA-G1	Crystal structure [56]
		sHLA-G1	Surface plasmon resonance [35]
		HLA-G tetramers	Tetramer-binding assays [55]
		sHLA-G	Cell binding assays [57]
KIR2DL4	NK cells	mHLA-G	Binding assays and cytotoxicity assays [36]
		mHLA-G	Cytotoxicity assays [58]
		mHLA-G	Binding assays [59]
CD8	CD8^+^ T cells	sHLA-G1	Apoptosis assay [60]
CD160	Endothelial cells	sHLA-G1	Radiolabeled cell-binding competition assay, tetramer-binding, antibody-blocking [39]
		mHLA-G	Conjugate formation (cell binding assay) of CHO-CD160 transfectants and HLA-expressing cells [61]

**Table 2:** Summary of statistical values of the identified receptor candidates and ligands.

Protein		Log2FC	−Log10(adj. *p*-Value)
MOT2	Monocarboxylate transporter 2	−2.95164	2.948745312
S43A3	Solute carrier family 43 member 3	−1.19412	3.325027107
MOT1	Monocarboxylate transporter 1	−1.8302	5.028116407
PPIB	Peptidyl-prolyl cis-trans isomerase	1.274108	7.824816967
MK	Midkine	5.897202	10.51019622
B2MG	β_2_m	2.770848	11.09377281
TFR1	Transferrin receptor protein 1	−1.64478	12.72159366
MOT4	Monocarboxylate transporter 4	−2.89668	12.72159366
HLAG	HLA-G	4.550522	12.72159366
TRFE	Transferrin	−6.51903	12.72159366

## Abbreviations

AA	amino acid
ß_2_m	β2-microglobulin
DC	dendritic cell
HLA	human leukocyte antigen
HSCT	hematopoietic stem cell transplantation
ITIMs	immune receptor tyrosine-based inhibitory motifs
NK cell	natural killer cell
mHLA-G	membrane-bound HLA-G
sHLA-G	soluble HLA-G
sNKG2A/CD94	soluble NKG2A/CD94
sNKG2C/CD94	soluble NKG2C/CD94

## Appendix A

### Native PAGE for Verification of Heterodimeric Complex of NKG2A/CD94 and NKG2C/CD94

In a native PAGE, proteins are separated in their natural conformation according to their charge to mass ratio. Purified protein was mixed 1:2 with *Novex*^®^ Tris-Glycine Native Sample Buffer (Invitrogen, Carlsbad, CA, USA) and loaded on a *Novex*^®^ 4–12% Tris-Glycine Protein Gel (Invitrogen, Carlsbad, CA, USA) in *Novex*^®^ Tris-Glycine Native Running Buffer (Invitrogen, Carlsbad, CA, USA). NativeMark™ Unstained Protein Standard (Invitrogen, Carlsbad, CA, USA) was used as marker. Gel was run for 2.5 h at 125 V.
